# Supplementary material for: A multiethnic genome-wide analysis of 44,039 individuals identifies 41 new loci associated with central corneal thickness
Source: Commun Biol. 2020 Jun 11;3:301. doi: 10.1038/s42003-020-1037-7 (PMC7289804; doi:10.1038/s42003-020-1037-7)
Supplement: Supplementary file 1 — Supplementary Information [file 42003_2020_1037_MOESM1_ESM.pdf]

Supplementary Information for “A multiethnic genome-wide analysis of 44,039 individuals identifies 41 new loci associated with central corneal thickness”

Choquet et al.

Supplementary Figure 1. QQ plot and genomic inflation factors (λ) observed for the GERA non-Hispanic whites GWAS of CCT

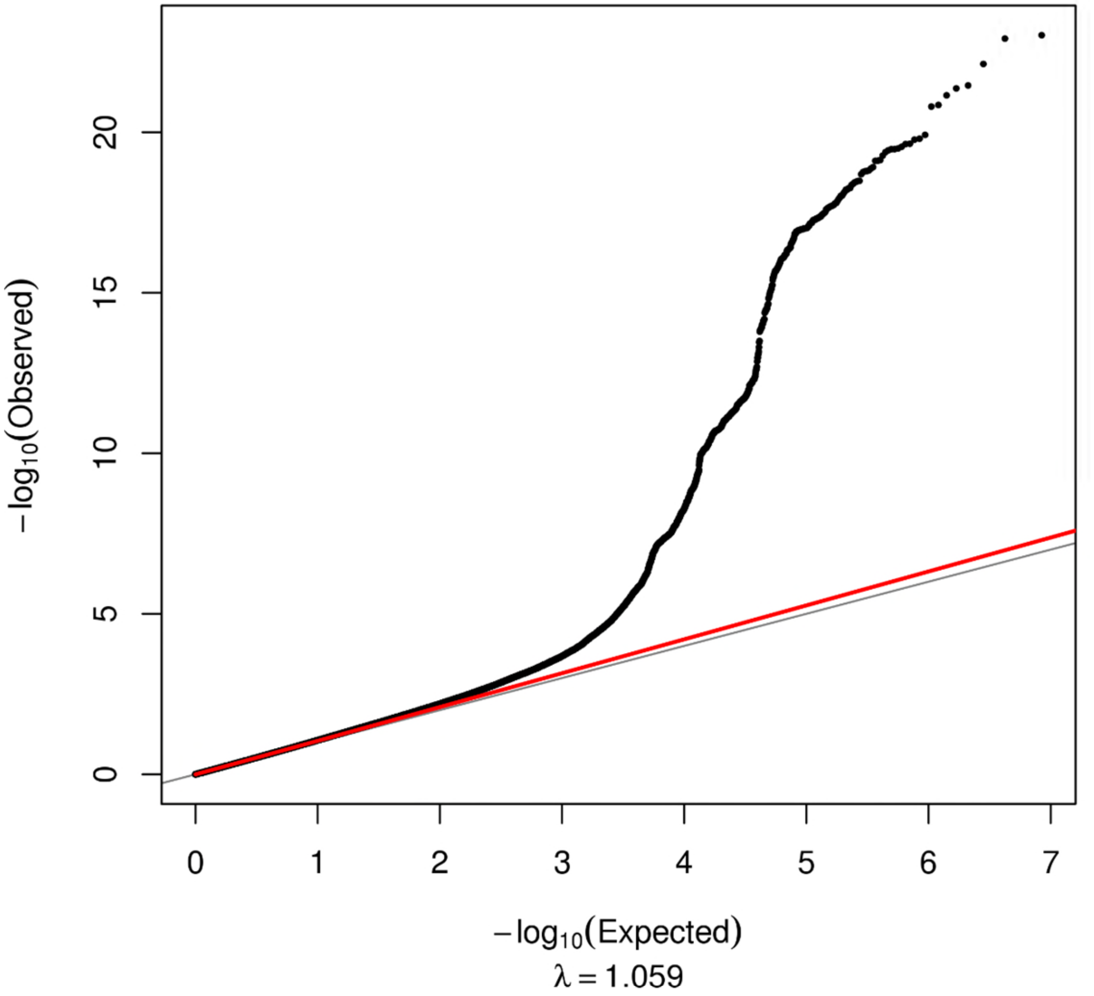

Supplementary Figure 2. QQ plot and genomic inflation factors (λ) observed for the GERA Hispanic/Latinos GWAS of CCT

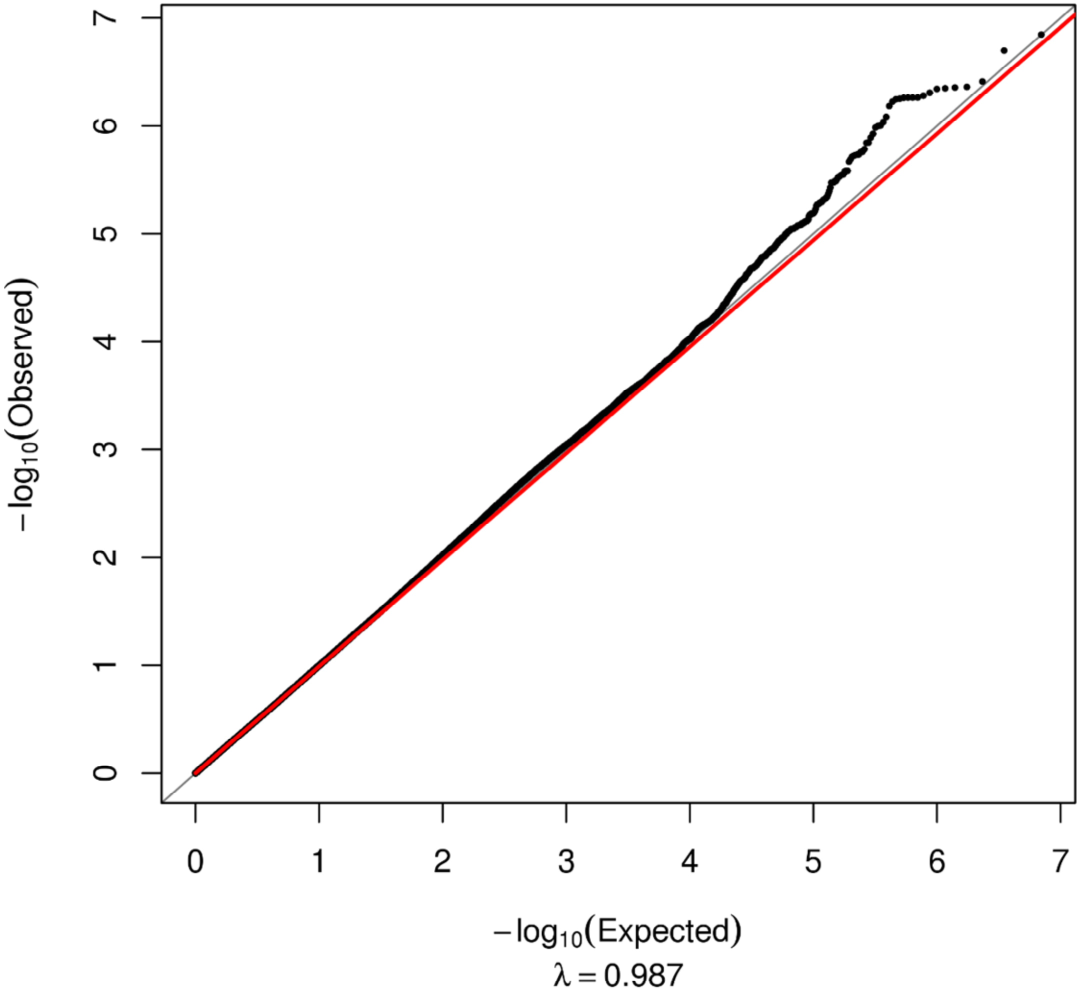

Supplementary Figure 3. QQ plot and genomic inflation factors (λ) observed for the GERA East Asians GWAS of CCT

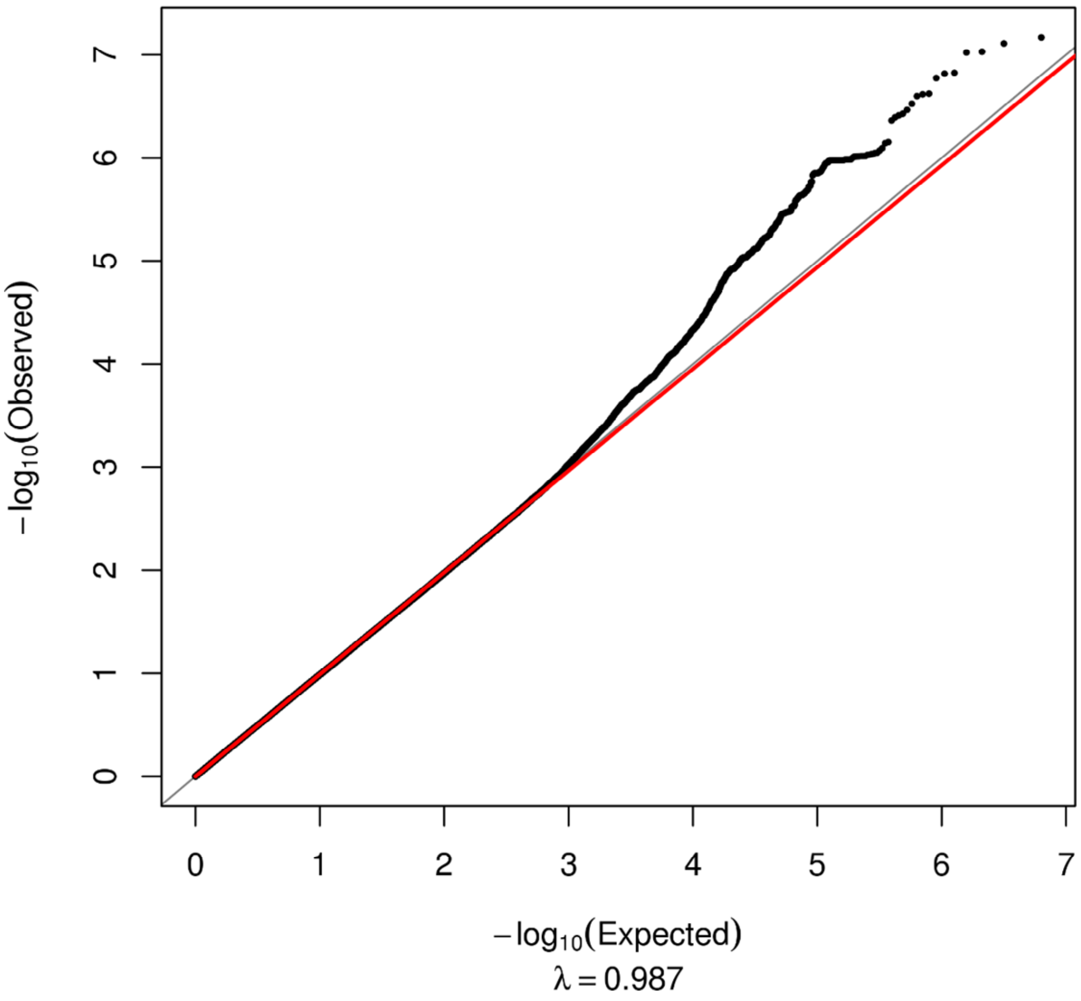

Supplementary Figure 4. QQ plot and genomic inflation factors (λ) observed for the GERA African Americans GWAS of CCT

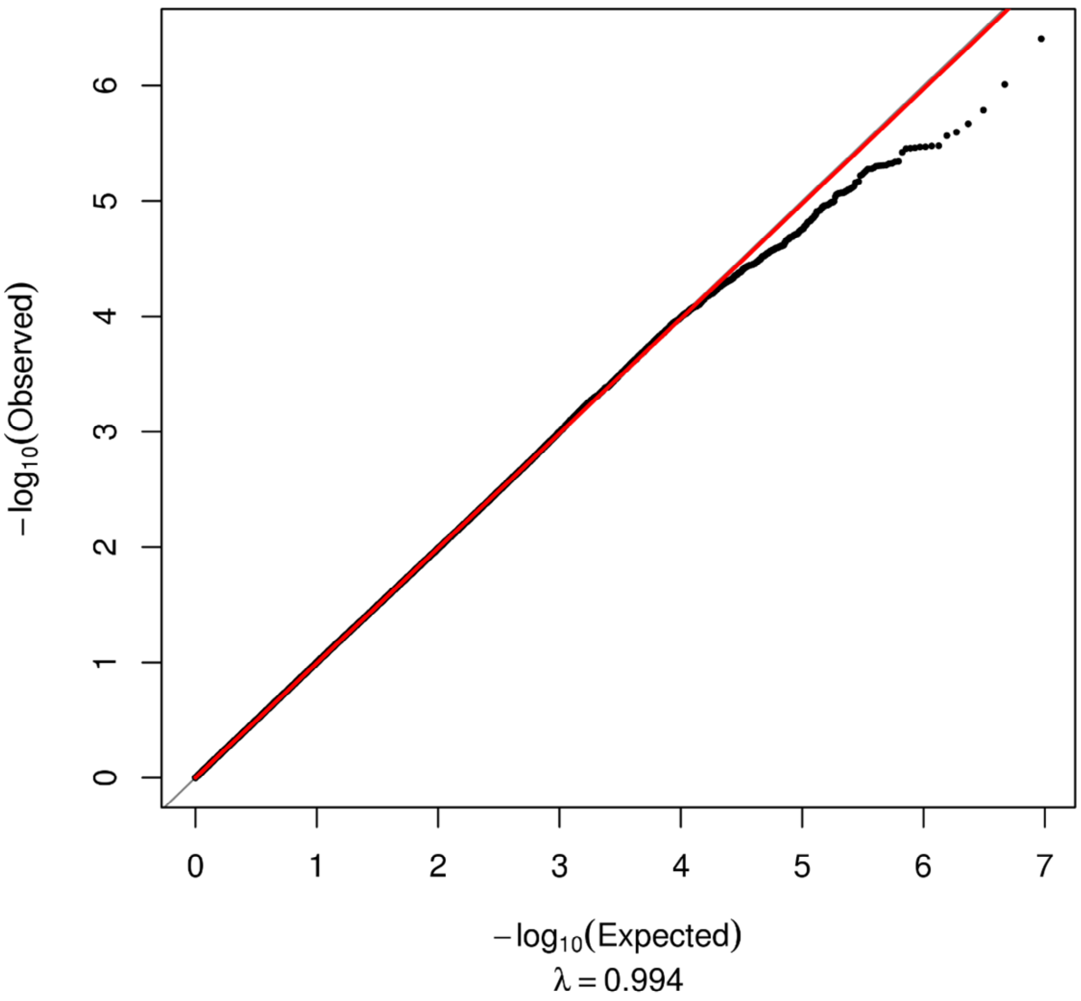

Supplementary Figure 5. QQ plot and genomic inflation factors (λ) observed for the GERA multiethnic meta-analysis of CCT

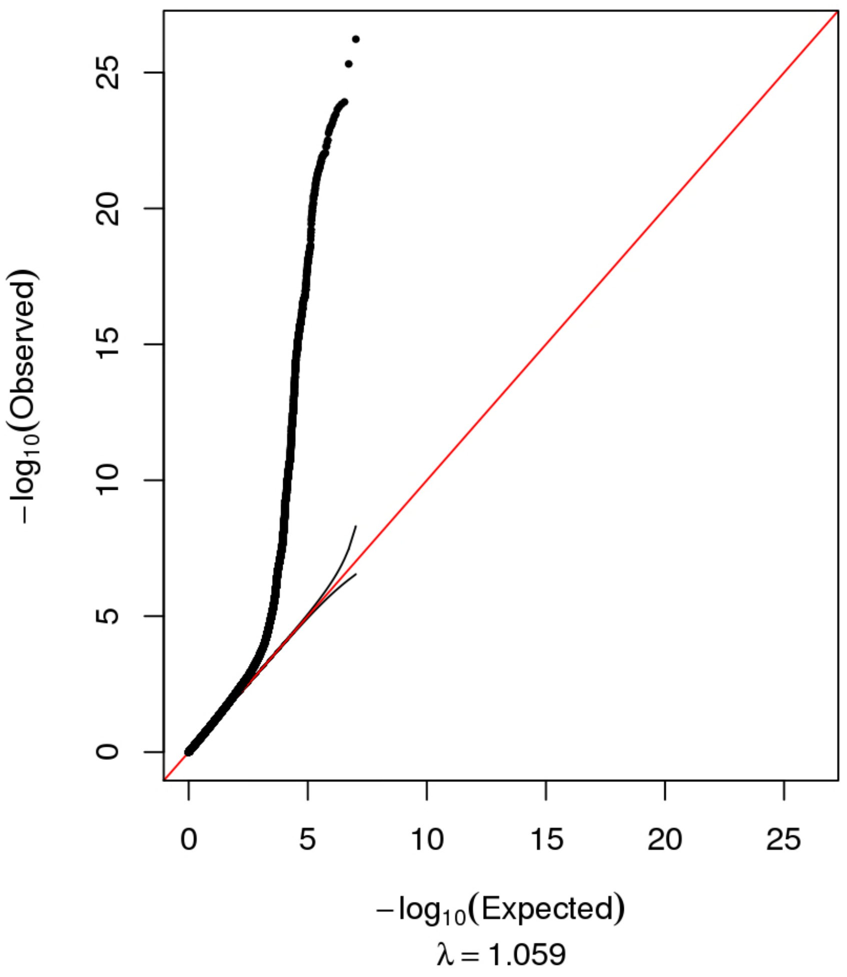

Supplementary Figure 6. QQ plot and genomic inflation factors (λ) observed for the combined (GERA+IGGC) multiethnic meta-analysis of CCT

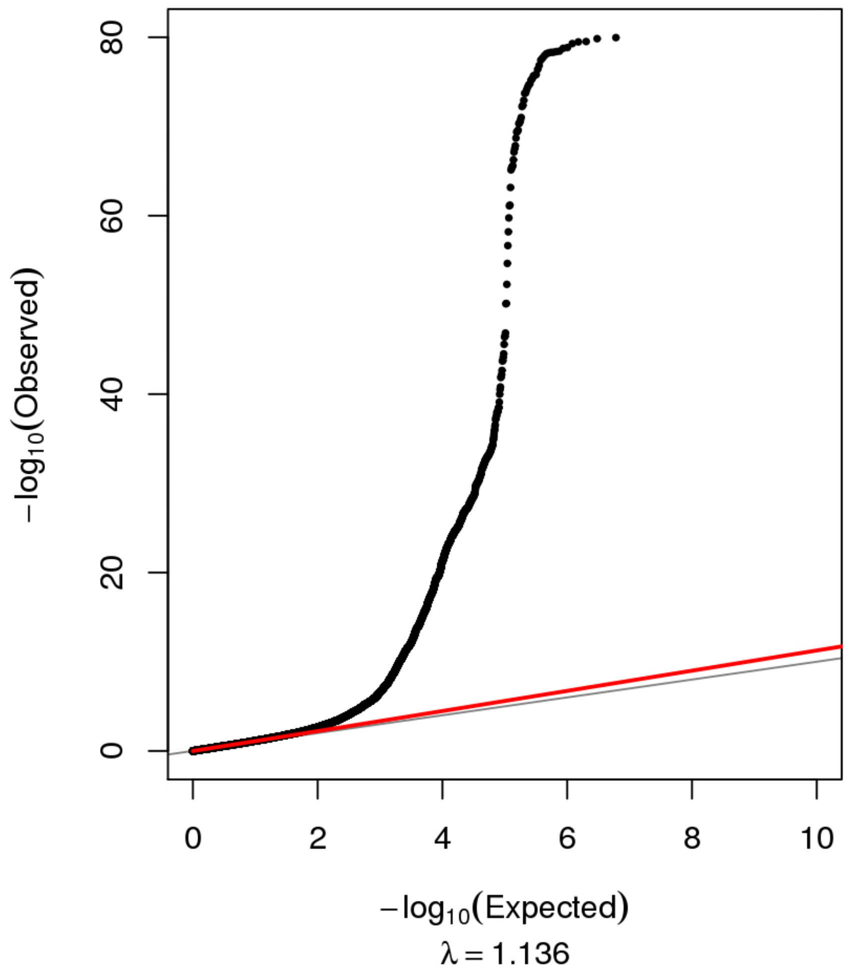

Supplementary Figure 7. Chicago plot of the GWAS meta-analyses of CCT in European-specific samples (upper panel) and Asian-specific samples (lower panel)

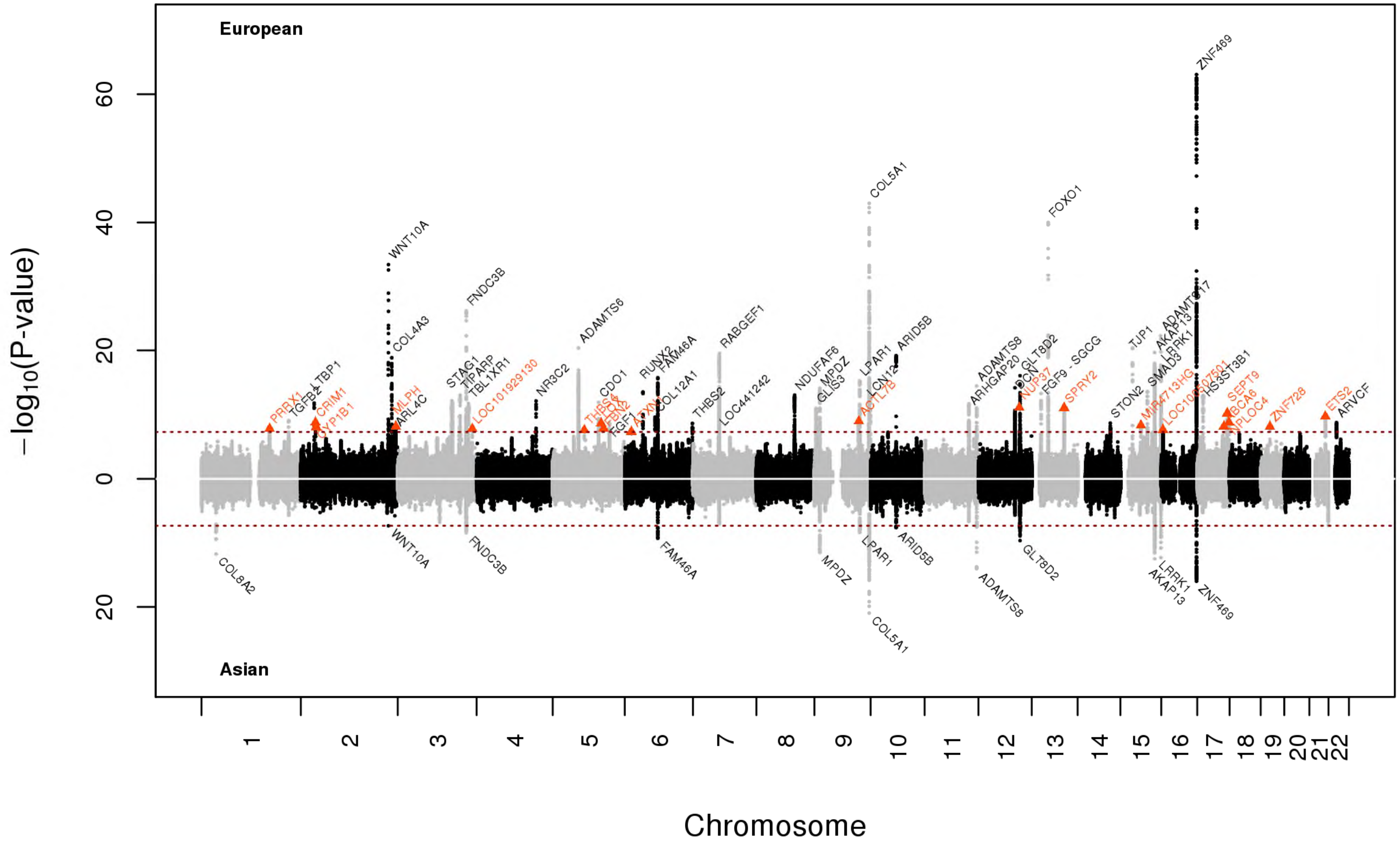

Association results (–log10 P-values) are plotted for each chromosome. The locus/gene names are indicated in black for previously reported loci, and in orange for the novel loci identified in the combined (GERA+IGGC) meta-analysis.

Supplementary Figure 8: Correlation of effect estimates between CCT and keratoconus. Each dot represents one of the 98 lead CCT-associated SNPs. CCT beta values are from the combined (GERA + IGGC) meta-analysis. In red, SNPs that surpassed the Bonferroni-corrected significance threshold (*P*<5.2x10^{-4}) in the keratoconus (GERA + independent cohort from the UK) meta-analysis. In blue, SNPs that were associated at a nominal level of significance (*P*<0.05) in the keratoconus (GERA + independent cohort from the UK) meta-analysis.

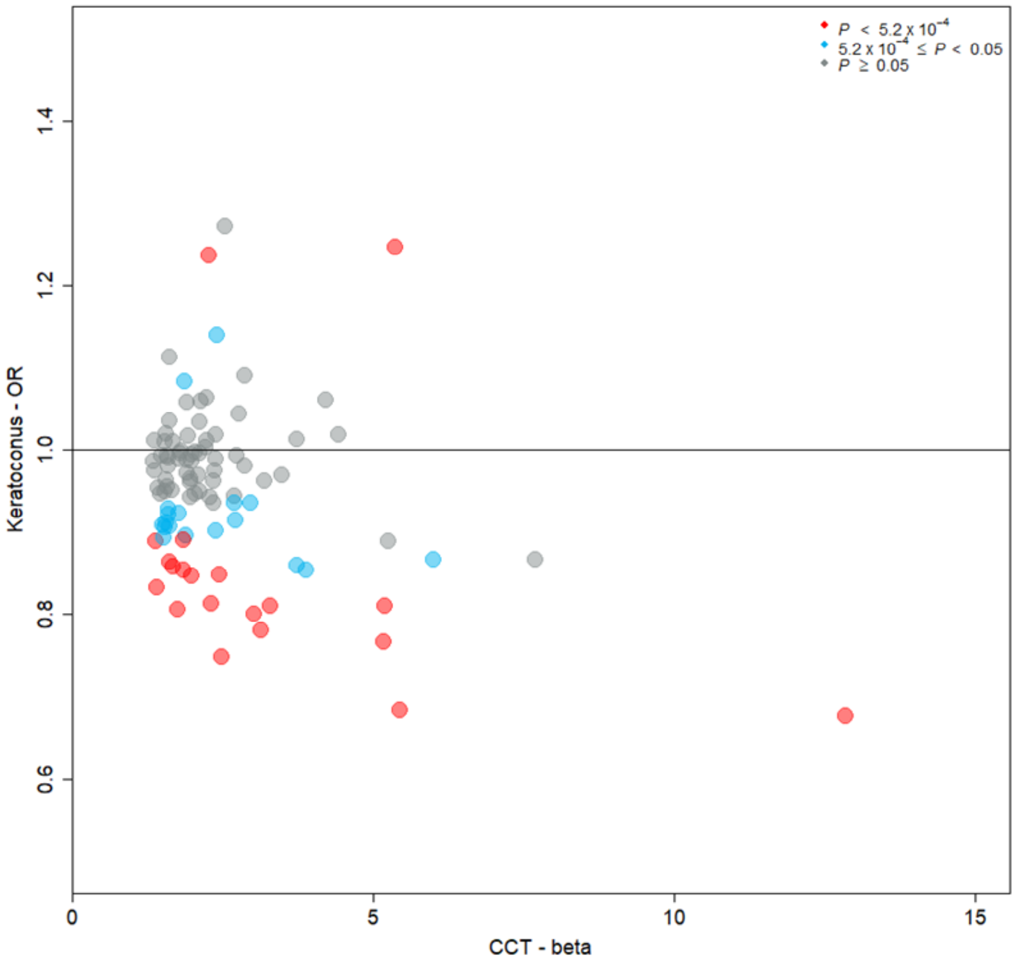

Supplementary Figure 9: Correlation of effect estimates between CCT and POAG. Each dot represents one of the 98 lead CCT-associated SNPs. CCT beta values are from the combined (GERA + IGGC) meta-analysis. In red, SNPs that surpassed the Bonferroni-corrected significance threshold ($P<5.2\text{x}10^{-4}$), including *RAPSN* rs3740685 in the GERA POAG analysis. In blue, SNPs that were associated at a nominal level of significance ($P<0.05$) in the POAG GERA analysis.

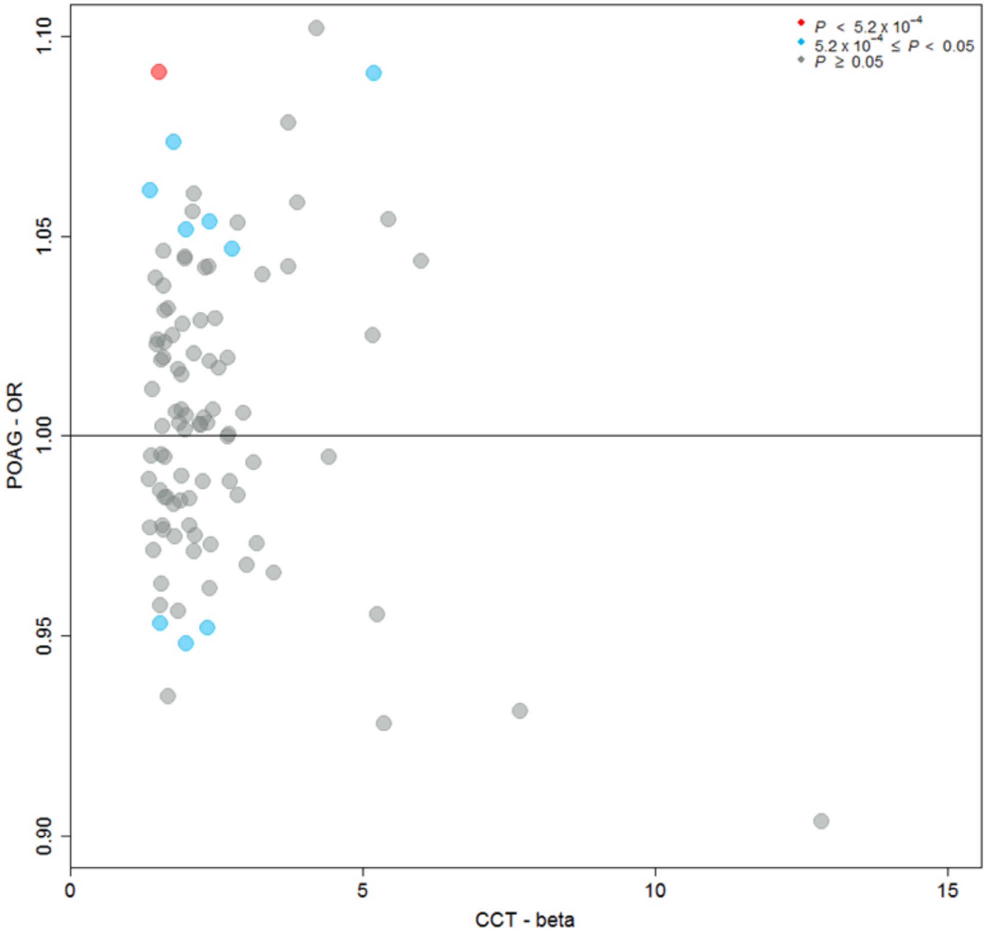

Supplementary Figure 10. Regional association plots of the *RAPSN* locus based on GERA meta-analysis results. Regional association plots for *RAPSN* locus with 400 kb upstream and downstream regions show: (**a**) the association with CCT and (**b**) the association with POAG for SNPs located at the *RAPSN* locus.

a.

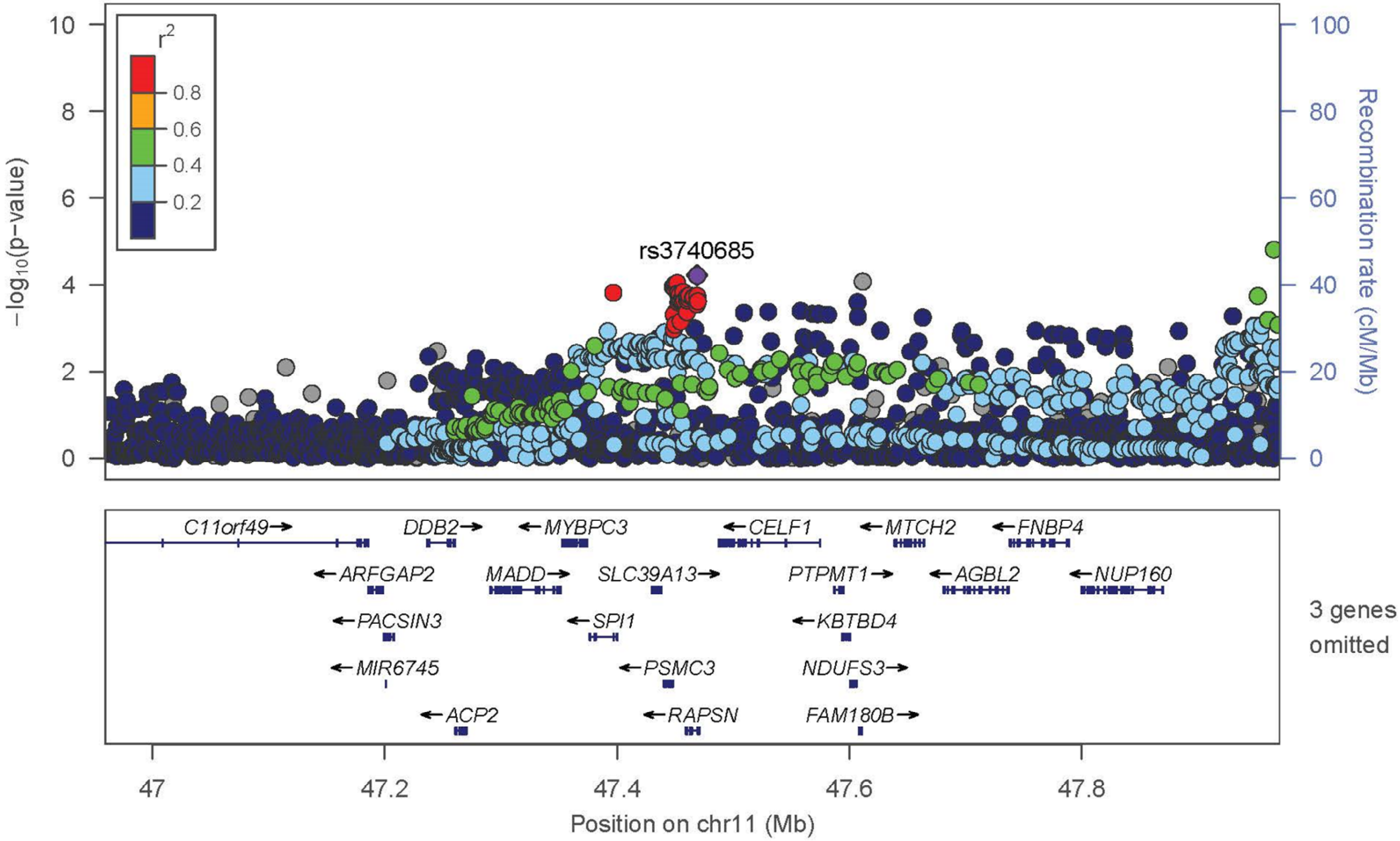

b.

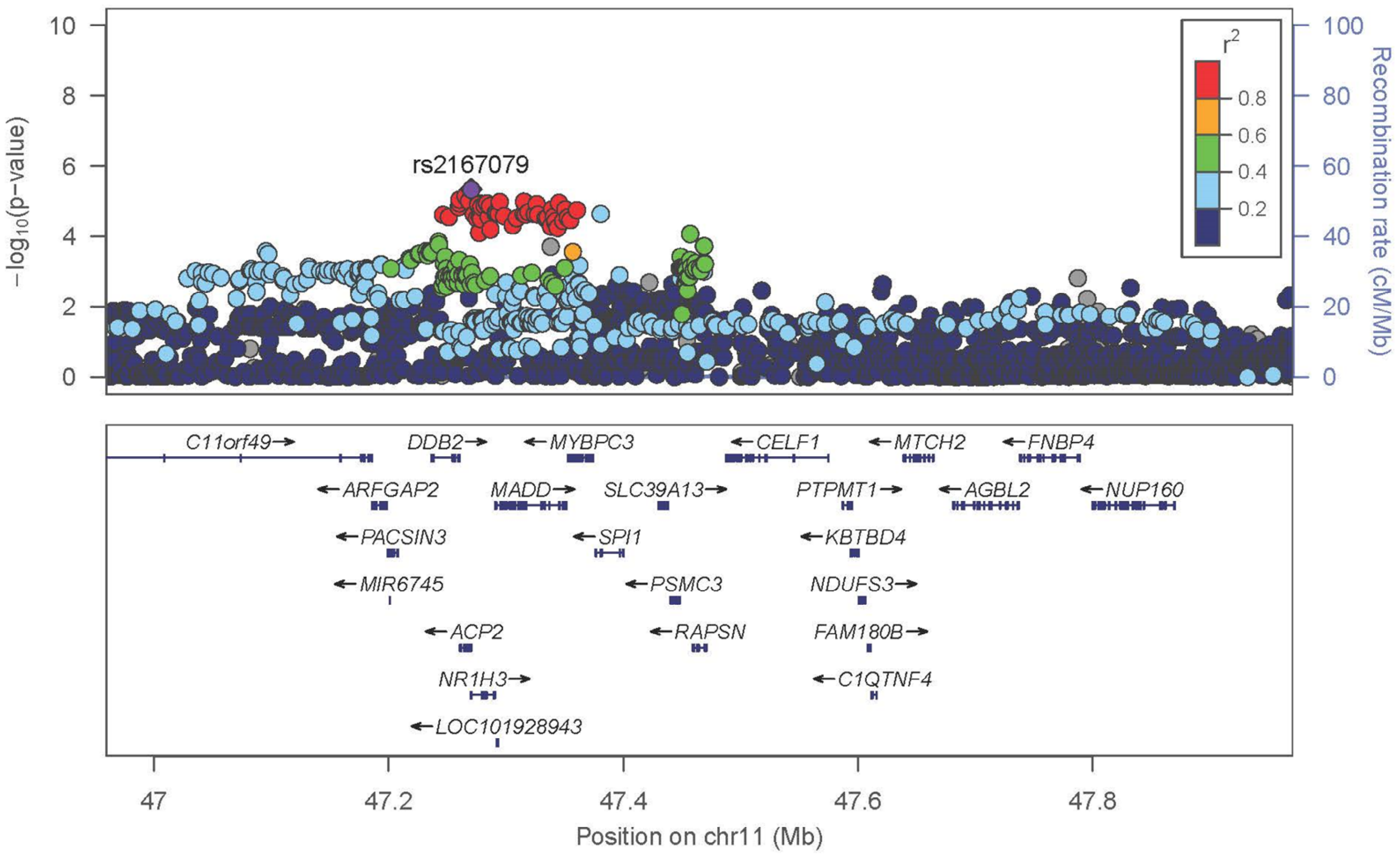

rs2167079
r2
0.8
0.6
0.4
0.2
−log10(p−value)
10
8
6
4
2
0
100
80
60
40
20
0
Recombination rate (cM/Mb)
C11orf49
DDB2
MYBPC3
CELF1
MTCH2
FNBP4
ARFGAP2
MADD
SLC39A13
PTPMT1
AGBL2
NUP160
PACSIN3
SPI1
KBTBD4
MIR6745
PSMC3
NDUFS3
ACP2
RAPSN
FAM180B
NR1H3
C1QTNF4
LOC101928943
47
47.2
47.4
47.6
47.8
Position on chr11 (Mb)

Supplementary Figure 11: Mendelian randomization plots for relationship of CCT with POAG

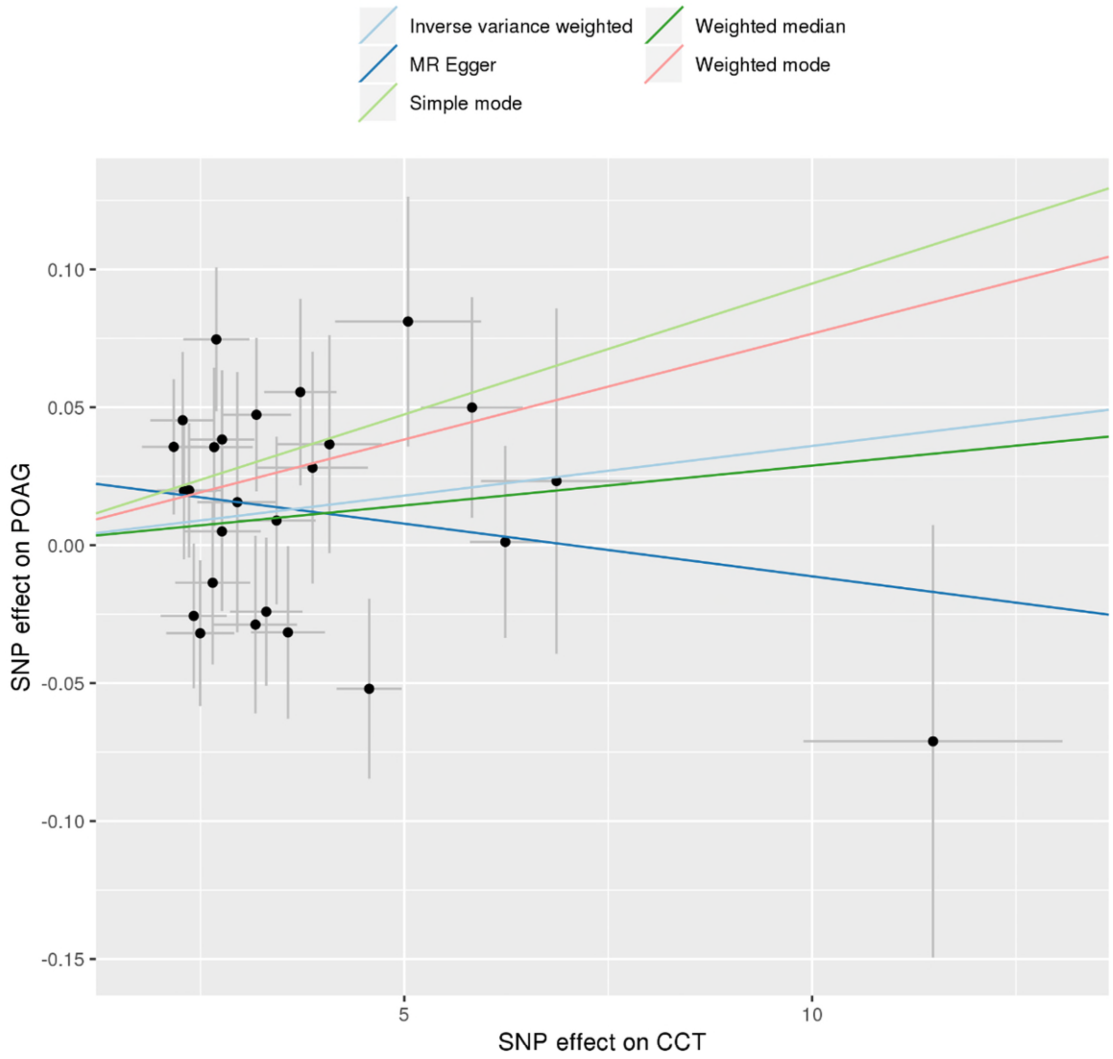

Supplementary Figure 12: Mendelian randomization leave-one-out analysis for CCT suggesting no evidence of causality with POAG. MR estimates using the inverse variance–weighted (IVW) method are reported.

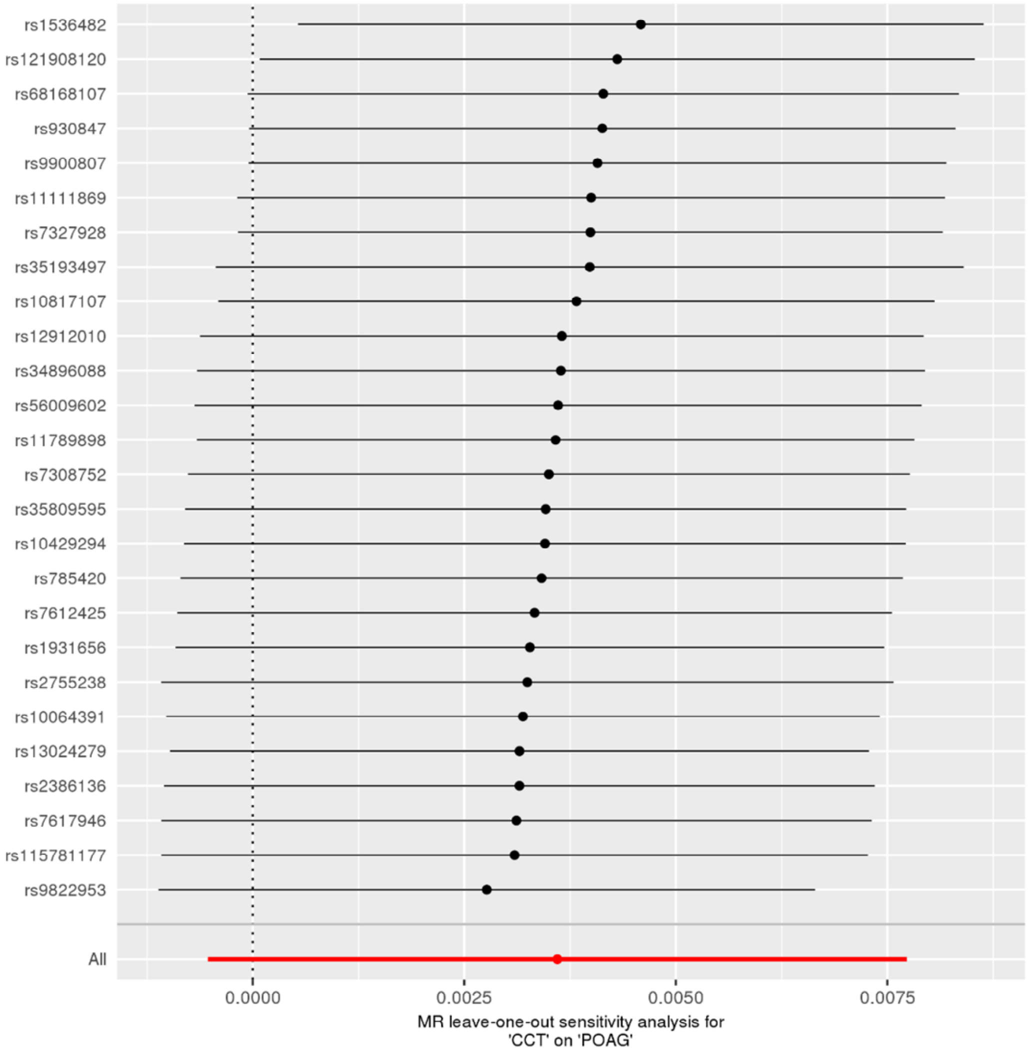

Supplementary Figure 13: Mendelian randomization single-SNP-only sensitivity analysis for CCT suggesting no evidence of causality with POAG

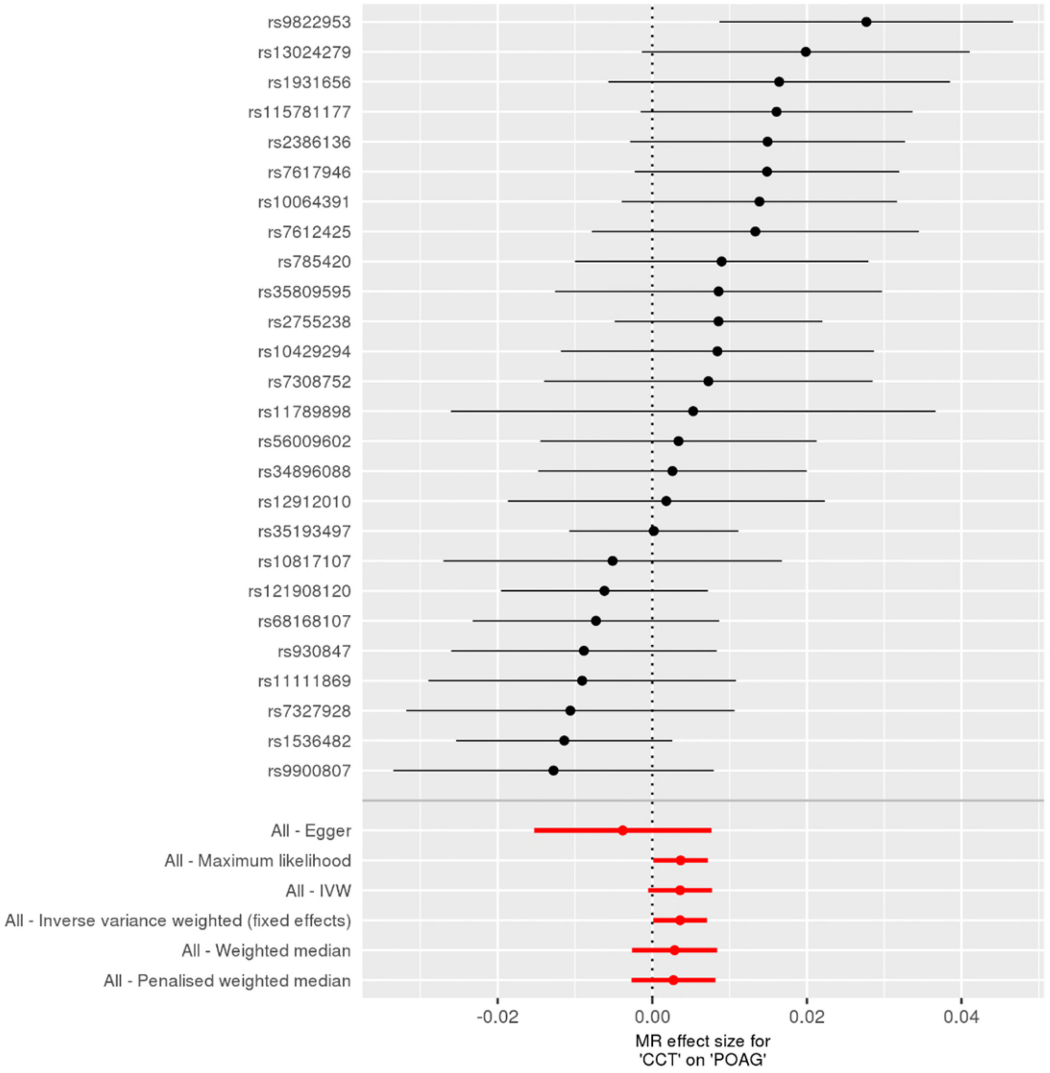

Supplementary Figure 14: Comparison between left (OS) and right (OD) CCT measures for each GERA participant included in the current study

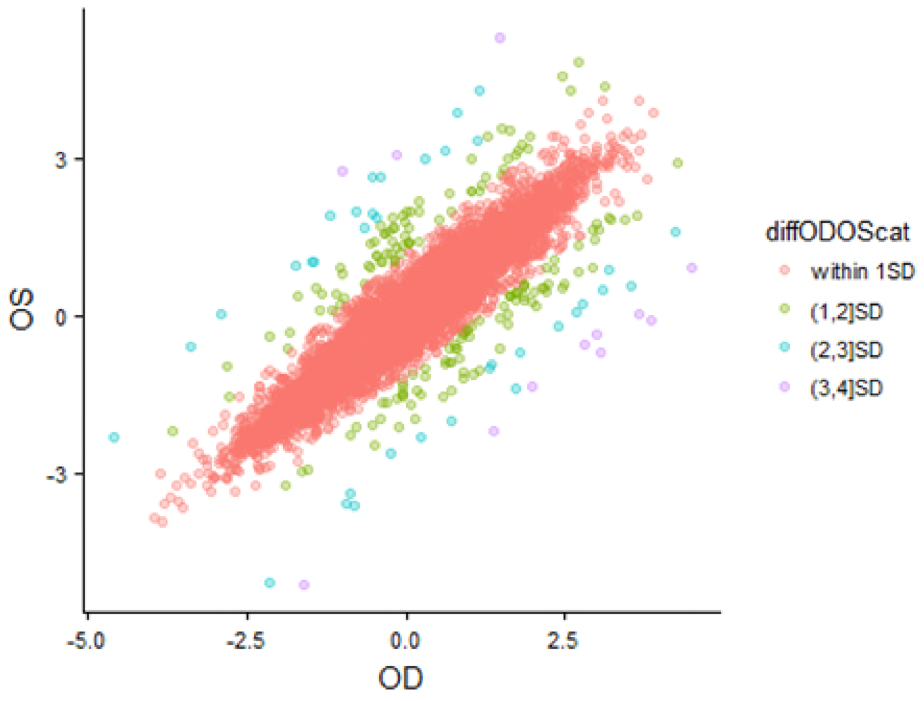

Outliers (N=9) defined by large left-right differences (i.e., beyond 4 sd of the overall standardized distribution of left-right differences) were removed
