# Supplementary material for: A multiethnic genome-wide analysis of 44,039 individuals identifies 41 new loci associated with central corneal thickness
Source: Commun Biol. 2020 Jun 11;3:301. doi: 10.1038/s42003-020-1037-7 (PMC7289804; doi:10.1038/s42003-020-1037-7)
Supplement: Supplementary file 2 — Description of Additional Supplementary Files [file 42003_2020_1037_MOESM2_ESM.pdf]

**Supplementary Data**

**Supplementary Data 1.** Association of each covariate with CCT in the GERA cohort

Covariates include age, sex, type of measurement (i.e., ultrasonic pachymeter and non-contact optical biometer) and ancestry PCs. Abbreviations: PC, principal component; ASHK, Ashkenazi ancestry proportion; β, beta; SE, standard error. For the interpretation of the PCs, see Banda et al. Genetics 2015. Effect size estimates (β) and p-values are from the multivariate linear regression model.

**Supplementary Data 2.** Lead CCT-associated SNPs previously identified in the IGGC study and look-up in the GERA samples cohort

**Supplementary Data 3.** Lead CCT-associated SNPs identified in the GERA meta-analysis and look-up in the IGGC study

**Supplementary Data 4.** CCT-associated loci (novel and previously reported) identified in the combined meta-analysis (GERA+IGGC) and look-up in the individual samples

Loci in bold are novel. Abbreviations: SNP, single-nucleotide polymorphism; Chr, chromosome, Pos position; β, beta; NA, not available.

**Supplementary Data 5.** CCT-associated variants identified in the conditional and joint multiple-SNP (COJO) analysis conducted on the combined (GERA+IGGC) meta-analysis results

In bold are novel independent CCT-associated SNPs. Linkage disequilibrium (LD) metrics (R2 and D') have all been calculated in European-ancestry populations using a web-based bioinformatic tool (https://analysistools.nci.nih.gov/LDlink/). Abbreviations: P, P-value from COJO analyses; NA, not available in 1000G reference panel; [a]index SNP associated with CCT from COJO analyses; [b]SNPs associated with CCT in previous studies.

**Supplementary Data 6.** Comparison of effect estimates of the 98 CCT lead SNPs between GERA non-Hispanic whites and GERA Hispanic/Latinos

**Supplementary Data 7.** Proportion of variance in CCT explained by all the 98 CCT-associated SNPs identified in the current study

The 98 CCT-associated SNPs include the 74 SNPs identified in the combined (GERA+IGGC) meta-analysis and the 24 from the COJO conditional analysis.

**Supplementary Data 8.** List of the 95% credible set of variants in each of the 74 CCT loci

**Supplementary Data 9.** DEPICT Gene Prioritization Results

**Supplementary Data 10.** Expression of the genes within the 74 CCT loci that contained associated 95% credible set variants in adult human eye tissues

Here, we included genes within the 74 CCT loci that contained associated 95% credible set variants. In the Ocular Tissue Database (OTDB), the gene expression is indicated as Affymetrix Probe Logarithmic Intensity Error (PLIER) number. The PLIER numbers were calculated by GC-background correction, PLIER normalization, log transformation and z-score calculation. The OTDB is available at https://genome.uiowa.edu/otdb/. In the EyeSAGE datasets from NEIBank, the gene expression is determined by tag counts in the Serial Analysis of Gene Expression (SAGE). All counts were summarized for each gene per tissue, and we added '+' to indicate the expression, or '-' to label the no expression while the counts are 0. The EyeSAGE is publicly available at the http://neibank.nei.nih.gov/EyeSAGE/index.shtml. Genes in bold are within novel CCT loci. PLIER numbers highlighted in yellow are >200 and for genes within novel CCT loci. Abbreviations: RPE, retinal pigment epithelium; TM, trabecular meshwork; MAC, retina macular; RPE Peri, RPE peripheral; na, not applicable.

**Supplementary Data 11.** DEPICT Tissue Enrichment

**Supplementary Data 12.** DEPICT Gene-set Enrichment

**Supplementary Data 13.** VEGAS2-Pathways association analysis top results

As 9,732 pathways or gene-sets from the Biosystem's database were tested, the P-value adjusted for Bonferroni correction was set as P<5.14x10-6 (0.05/9,732); pathways or gene-sets that reached this Bonferroni-level of significance are highlighted in grey.

**Supplementary Data 14.** Association between the identified 98 CCT-associated SNPs and keratoconus in the combined (GERA + independent keratoconus cohort) meta-analysis

**Supplementary Data 15.** Association between the identified 98 CCT-associated SNPs and POAG in GERA and confirmation for glaucoma in UKB

Analysis is adjusted for age at specimen, sex, ethnic group, and CCT measurement type (i.e., ultrasonic pachymeter and non-contact biometer).

**Supplementary Data 16.** Association between CCT (observational measure) and POAG status in GERA

**Supplementary Data 17.** Two-sample Mendelian Randomization Results for the Relationship Between CCT and POAG
