# Supplementary material for: A multiethnic genome-wide analysis of 44,039 individuals identifies 41 new loci associated with central corneal thickness
Source: Commun Biol. 2020 Jun 11;3:301. doi: 10.1038/s42003-020-1037-7 (PMC7289804; doi:10.1038/s42003-020-1037-7)
Supplement: Supplementary file 4 — Reporting Summary [file 42003_2020_1037_MOESM4_ESM.pdf]

nature research

Corresponding author(s): Hélène Choquet
Last updated by author(s): 5/19/2020

Reporting Summary

Nature Research wishes to improve the reproducibility of the work that we publish. This form provides structure for consistency and transparency in reporting. For further information on Nature Research policies, see Authors & Referees and the Editorial Policy Checklist.

Statistics

For all statistical analyses, confirm that the following items are present in the figure legend, table legend, main text, or Methods section.

n/a	Confirmed	
☐	☒	The exact sample size (*n*) for each experimental group/condition, given as a discrete number and unit of measurement
☐	☒	A statement on whether measurements were taken from distinct samples or whether the same sample was measured repeatedly
☐	☒	The statistical test(s) used AND whether they are one- or two-sided<br>*Only common tests should be described solely by name; describe more complex techniques in the Methods section.*
☐	☒	A description of all covariates tested
☐	☒	A description of any assumptions or corrections, such as tests of normality and adjustment for multiple comparisons
☐	☒	A full description of the statistical parameters including central tendency (e.g. means) or other basic estimates (e.g. regression coefficient) AND variation (e.g. standard deviation) or associated estimates of uncertainty (e.g. confidence intervals)
☐	☒	For null hypothesis testing, the test statistic (e.g. *F*, *t*, *r*) with confidence intervals, effect sizes, degrees of freedom and *P* value noted<br>*Give P values as exact values whenever suitable.*
☒	☐	For Bayesian analysis, information on the choice of priors and Markov chain Monte Carlo settings
☐	☒	For hierarchical and complex designs, identification of the appropriate level for tests and full reporting of outcomes
☐	☒	Estimates of effect sizes (e.g. Cohen's *d*, Pearson's *r*), indicating how they were calculated

Our web collection on statistics for biologists contains articles on many of the points above.

Software and code

Policy information about availability of computer code

Data collection	Genotype analysis, quality control, phasing, and imputation analysis were performed on samples from the GERA cohort using the following softwares: Genotyping Console™ Software (Affymetrix) to perform genotype calling, quality control (QC) analysis, and sample or SNP filtering prior to downstream analysis. PLINK software v1.90 to perform additional QC analyses. Shape-IT v2.r727195861 to conduct phasing and IMPUTE2 v2.3.05962 to perform imputation. All software programs employed are available for public use and no custom code was employed.
Data analysis	Eigenstrat v4.2 was used to calculate the principal components (PCs) on each of the four GERA ethnic groups. PLINK v1.9 was used to perform a linear regression of the outcome and each SNP. Other statistic analyses and data management were performed in the language-and-environment R, version 3.6.0, using functions from the default libraries. Genome-wide Complex Trait Analysis (GCTA) integrative tool was used to conduct a multi-SNP-based conditional & joint association analysis (COJO), to estimate array-heritability, and to estimate the proportion of variance in our trait explained. CAVIARBF was used to prioritize genetic variants within the identified genomic regions. The DEPICT software was used to prioritize genes and biological pathways, and highlight gene-set and tissue/cell enrichments within the identified genomic regions.

For manuscripts utilizing custom algorithms or software that are central to the research but not yet described in published literature, software must be made available to editors/reviewers. We strongly encourage code deposition in a community repository (e.g. GitHub). See the Nature Research guidelines for submitting code & software for further information.

Data

Policy information about availability of data

All manuscripts must include a data availability statement. This statement should provide the following information, where applicable:

- Accession codes, unique identifiers, or web links for publicly available datasets
- A list of figures that have associated raw data
- A description of any restrictions on data availability

The GERA genotype data are available upon application to the KP Research Bank (https://researchbank.kaiserpermanente.org/). The summary statistics generated

in the study of Iglesias et al. are available at http://hdl.handle.net/10283/2976. The combined (GERA+IGGC) meta-analysis GWAS summary statistics are available from the NHGRI-EBI GWAS Catalog (https://www.ebi.ac.uk/gwas/downloads/summary-statistics).

Field-specific reporting

Please select the one below that is the best fit for your research. If you are not sure, read the appropriate sections before making your selection.

☒ Life sciences ☐ Behavioural & social sciences ☐ Ecological, evolutionary & environmental sciences

For a reference copy of the document with all sections, see nature.com/documents/nr-reporting-summary-flat.pdf

Life sciences study design

All studies must disclose on these points even when the disclosure is negative.

Sample size	Our study utilizes data from 44,039 individuals between the Genetic Epidemiology Research in Adult Health and Aging (GERA) cohort and the International Glaucoma Genetics Consortium (IGGC). In GERA, 18,129 participants from four ethnic groups (non-Hispanic white, Hispanic/Latino, East Asian, and African American) who had at least one recorded CCT measurement on both eyes during the same visit between June 2014 and January 2018 were included.
Data exclusions	GERA patients with single eye measurements were removed. We also excluded 1,106 patients who had ocular conditions which may influence CCT, including patients with Fuchs dystrophy, keratoconus, history of corneal refractive surgery, corneal transplantation, or laser vision surgery. Outliers (N=9) defined by large left-right differences (i.e., beyond 4 sd of the overall standardized distribution of left-right differences) were also removed.
Replication	The initial genome-wide association study, carried out in GERA was replicated in the IGGC and vice-versa (77% of genetic loci of interest replicated). Additionally, we assessed the correlation of effect sizes for CCT-associated SNPs (identified in the combined meta-analysis) between GERA and IGGC cohorts
Randomization	Allocation of participants was not random in this study. Participants were enrolled and then selected based on ongoing longitudinal records from vision examinations.
Blinding	Blinding was not relevant to our study. Participants were selected based on ongoing longitudinal records from vision examinations, and if they had CCT measurements recorded for both eyes in the electronic health records.

Reporting for specific materials, systems and methods

We require information from authors about some types of materials, experimental systems and methods used in many studies. Here, indicate whether each material, system or method listed is relevant to your study. If you are not sure if a list item applies to your research, read the appropriate section before selecting a response.

Materials & experimental systems

n/a	Involved in the study	
☒	☐	Antibodies
☒	☐	Eukaryotic cell lines
☒	☐	Palaeontology
☒	☐	Animals and other organisms
☐	☒	Human research participants
☐	☒	Clinical data

Methods

n/a	Involved in the study	
☒	☐	ChIP-seq
☒	☐	Flow cytometry
☒	☐	MRI-based neuroimaging

Human research participants

Policy information about studies involving human research participants

Population characteristics	The Genetic Epidemiology Research in Adult Health and Aging (GERA) cohort consists of 110,266 adult men and women, 18 years and older, who are of non-Hispanic white, Hispanic/Latino, Asian or African American ethnicity.
Recruitment	Participants from the GERA cohort are members of the Kaiser Permanente Northern California (KPNC) integrated health care delivery system, and provided self-reported information via the Research Program on Genes, Environment, and Health (RPGEH) survey.
Ethics oversight	All study procedures were approved by the Institutional Review Board of the Kaiser Permanente Northern California Institutional Review Board. Written informed consent was obtained from all participants.

Note that full information on the approval of the study protocol must also be provided in the manuscript.

Clinical data

Policy information about clinical studies

All manuscripts should comply with the ICMJE guidelines for publication of clinical research and a completed CONSORT checklist must be included with all submissions.

Clinical trial registration	NA
Study protocol	NA
Data collection	GERA participants included in this study had at least one recorded CCT measurement on both eyes during the same visit between June 2014 and January 2018.
Outcomes	Central corneal thickness (CCT) was measured in GERA using the DGH-550 or DGH-55 ultrasonic (contact) pachymeter (DGH Technology Inc.; Exton, PA), or a non-contact optical biometer (Lenstar LS900, Haag-Streit, Köniz, Switzerland), and recorded for both eyes in the electronic health records.
